# Protective effect of ethanolic extract of *Echinacea purpurea* contained nanoparticles on meniscal/ligamentous injury induced osteoarthritis in obese male rats

**DOI:** 10.1038/s41598-022-09380-w

**Published:** 2022-03-30

**Authors:** Athira Johnson, Yu-Chia Huang, Chien-Feng Mao, Chun-Kai Chen, Sabu Thomas, Hsiang-Ping Kuo, Song Miao, Zwe-Ling Kong

**Affiliations:** 1 Department of Food Science, National Taiwan Ocean University, Pei-Ning Road, Keelung City, 20224 Taiwan, ROC; 2 School of Energy Studies and School of Chemical Sciences, Mahatma Gandhi University, Priyadarshini Hills P.O, Kottayam, Kerala 686560 India; 3 Deep Research (Xiamen) Health Industry Co., Ltd. Zone B1, 5th Floor, Factory 1, 889 Xinmin Avenue, Tongan District, Xiamen, 361113 Fujian China; 4 Teagasc Food Research Centre, Moorepark, Fermoy, Co. Cork P61 C996 Ireland

**Keywords:** Drug discovery, Immunology, Rheumatology

## Abstract

Osteoarthritis (OA) is a chronic degenerative joint disease associated with age, mechanical stress, and obesity*. Echinacea purpurea* is a medicinal plant that shows good anti-inflammatory, antioxidant, and immunomodulatory activities. In this study, *Echinacea purpurea* ethanol extract nanoparticles (Nano-EE) were prepared by encapsulating *Echinacea purpurea* ethanol extract (EE) in chitosan-silica nanoparticles. Obesity (OB) in Sprague-Dawley (SD) rats was induced by fed 40% high-fat diet and then anterior cruciate ligament and meniscus injury were performed to induce OA. The rats got different doses of samples by oral gavage. The encapsulation efficiency and loading capacity of Nano-EE were 69.1% and 36.1%, respectively. The average size, polydispersity index (PDI), and zeta potential (ZP) of the Nano-EE were 145 ± 11 nm, 0.24 ± 0.01, − 4.57 ± 0.44 mV, respectively. Furthermore, electron microscopic images showed that the particles were spherical and were slightly agglomerated. Moreover, it showed that the leptin content, expression of MMPs, cytokines level, NF-κB level, and iNOS production were decreased whereas collagen II expression was increased after treatment. Besides, Nano-EE ameliorated the pain caused by OA and reduced the proteoglycan loss in cartilage. These results indicated that encapsulated EE (Nano-EE) can ameliorate OA with a low dosage and are more effective than unencapsulated EE.

## Introduction

OA is one of the most common chronic degenerative joint diseases that affect hundreds of millions of people all over the world^1^. OA is mainly caused by several factors including obesity, age, genetic factors, and mechanical stress. Recently, the incidence of OA is increased due to obesity and aging^2^. OA can cause cartilage hypertrophy, cartilage degeneration, meniscal tears, osteophyte, subchondral bone sclerosis, and synovitis erosion, etc. This may finally lead to obstacles in walking and affect the quality of a patient’s life^3^. The literature stated that an increased expression of proinflammatory factors such as tumor necrosis factor (TNF)-α, interleukin (IL)-1β, IL-6, and leptin content were observed in blood under obese conditions^4^. Overexpression of TNF-α and IL-1β activates the nuclear factor-κB (NF-κB) pathway and produce matrix metalloproteinases (MMP), such as MMP-1, MMP-3, MMP-10, MMP-12, and MMP-13^5^. MMP is an enzyme involved in the degradation of the extracellular matrix and related to the breakdown of articular cartilage^6^. The extracellular matrix is composed of type II collagen and proteoglycan^7^. As a result of increased joint loading under the obese condition, cartilage tissue produce an inflammatory response, and this may result in the production of MMP and proinflammatory factors^8^.

However, the drugs currently used for OA treatment including non-steroidal anti-inflammatory drugs (NSAID) increase the risk of kidney damage and cardiovascular disease^9^. *Echinacea purpurea* is a medicinal plant native to North America. Literature mentioned that, it can be used for the treatment of wounds, burns, insect bites, toothache, snake bites, skin diseases, epilepsy, etc. Besides, it helps to improve the immune functions of the body^10^. It is known that, *Echinacea purpurea* has immunoregulatory^11^, antioxidant^12^, anti-inflammatory^13,14^, antibacterial^15^, antiviral^16^, antiproliferative^17^, hypoglycemic^17^, antihypertensive^18^, and anti-obesity^19^ activities, etc. Literature mentioned that alkylamide, caffeic acid derivatives, and polysaccharides are the major functional components found in Echinacea^20^. However, these functional ingredients are associated with poor water solubility, inconsistent oral bioavailability, and ease of dilution by digestive juice in the gastrointestinal tract^21^. To solve this problem, the plant extract was encapsulated in chitosan silica nanoparticles. Encapsulation is the process of using a drug carrier system to protect and deliver active compounds into the targeted sites. Microscale encapsulation is known as microencapsulation (encapsulating an active ingredient in particle size of 1–1000 µm in diameter) and nanoscale encapsulation is known as nanoencapsulation (encapsulating an active ingredient in particle size of 1–1000 nm in diameter)^22^. Encapsulation of functional components with micro-nanoencapsulation technology can improve the water solubility of the sample and prolong the release time of the therapeutic agent in the gastrointestinal tract, and thereby avoiding the dilution and decomposition of functional ingredients early by digestive juice^23^. Some studies have pointed out that in the weak acidic environment, chitosan and sodium silicate are polymerized to form chitosan-silica nanoparticles. Because positively charged chitosan is dissolved in an acid medium and combines with negatively charged silica to form chitosan-silica nanoparticles^24,25^.

Anterior cruciate ligament transection (ACLT) will cause the knee joint to lose its ability. Partial or complete removal of the meniscus will destroy the knee joint's ability to bear weight, and increase cartilage wear, and finally result in OA. Thus, ACLT and medial meniscectomy (ACLT + MMx) was used to induce OA in rats^26,27^. Therefore, this study investigated the use of *Echinacea purpurea* ethanol extract (EE) encapsulated chitosan silica nanoparticle to reduce and improve the OA condition (ACLT + MMx) in an animal model with high-fat diet-induced obesity)^25^. Previous studies reported the amelioration of inflammation and reproductive dysfunction in the presence of EE extract under obesity and diabetic conditions. However, it lacks to check the effect of EE extract under OBOA conditions^28,29^. Mao et.al. revealed that micro-nano encapsulated *Echinacea purpurea* ethanol extract has a size of about 218 nm. It was higher when compared to the current study^29^. The use of *Echinacea angustifolia* extract was found beneficial for knee OA and another study examined that the cichoric acid from Echinacea has anti-inflammatory effects over arthritis^30^. In comparatively, the current study focuses on the effects of encapsulated Echinacea extract over OA under obese conditions and it gives ideas of anti-inflammatory effects of the encapsulated extract.

## Results

### Sample analysis

#### Analysis of Nano-EE by high- performance liquid chromatography (HPLC)

**Figure 1:**
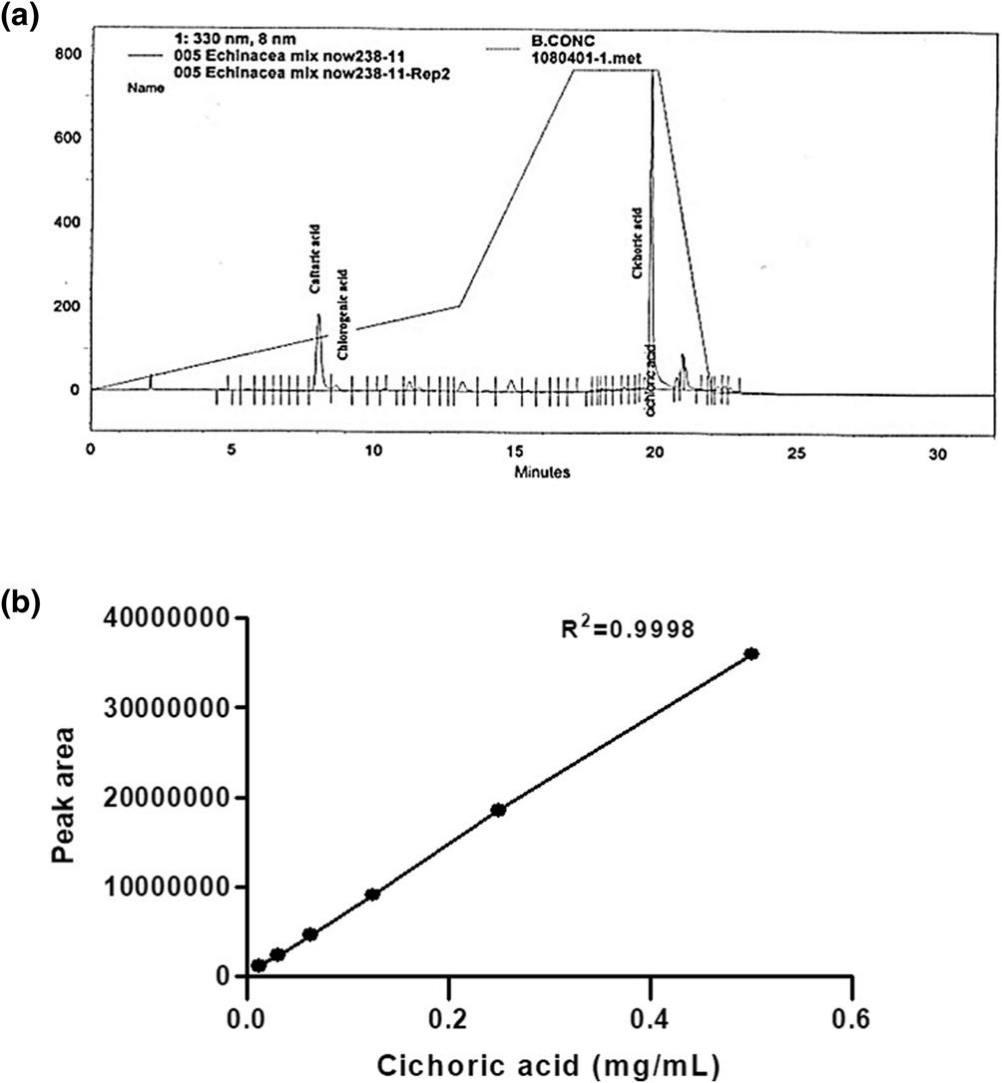
Determination of (**a**) total phenolic compounds in EE and (**b**) standard curve of cichoric acid.

The content of phenolic acid was determined by HPLC analysis (Fig. 1a, b). The standard curve was drawn from cichoric acid and the obtained R^2^ value of cichoric acid was 0.9998. The cichoric acid was selected because of it has a sharp peak obtained from HPLC analysis. Encapsulation efficiency (EE%) and loading capacity (LC%) of Nano-EE were calculated from the absorption peak of cichoric acid. The EE and LC of Nano-EE were 69.1% and 36.1%, respectively.

#### Analysis of particle size (PS), poly dispersity index (PDI), and zeta potential (ZP) of Nano-EE by dynamic light scattering (DLS)

DLS method was used to evaluate the size, PDI, and zeta potential of the Nano-EE (Table 1). The average size, PDI, and ZP of the Nano-EE were 145 ± 11 nm, 0.24 ± 0.01, − 4.57 ± 0.44 mV, respectively. After 3 months, the above said parameters were again checked and assessed for stability of the particle. It was found that the particle size became 234 ± 14 nm, the PDI value was 0.26 ± 0.03, and the zeta potential was − 4.39 ± 0.62 mV. Literature mentioned that the most suitable range of PDI is < 0.5^31^. The stability of the particle was again checked after 6 months and the obtained particle size was 352 ± 18 nm, the PDI value was 0.44 ± 0.04, and the zeta potential was − 3.76 ± 0.39 mV.

**Table 1:** Determination of particle size, polydispersity index (PDI), zeta potential (ZP), and stability (after 3 and 6 months) of Nano-EE by dynamic light scattering (DLS).

	PS (nm)	PDI	ZP (mV)
Nano-EE	145 ± 11	0.24 ± 0.01	− 4.57 ± 0.44
Nano-EE (after 3 months)	234 ± 14	0.26 ± 0.03	− 4.39 ± 0.62
Nano-EE (after 6 months)	352 ± 18	0.44 ± 0.04	− 3.76 ± 0.39

**Figure 2:**
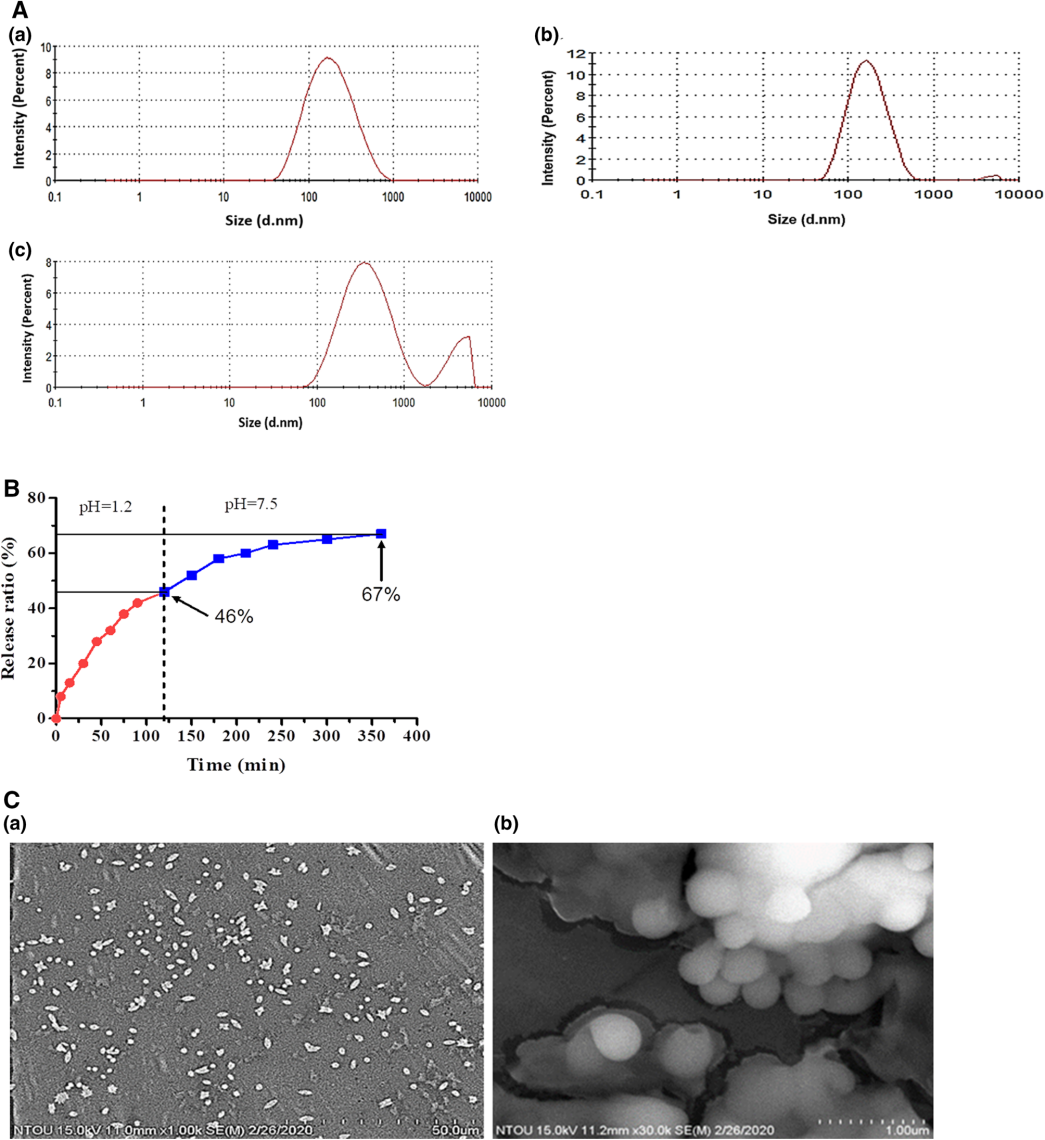
(**A**) Determination of (**a**) particle size distribution, (**b**) stability (after 3 months), and (**c**) stability (after 6 months) of Nano-EE by dynamic light scattering (DLS), (**B**) Drug releasing profiles of EE from Nano-EE in simulated gastric fluid (SGF) (red line) (pH = 1.2) and simulated intestinal fluid (SIF) (blue line) (pH = 7.5) by HPLC analysis, and (**C**) Scanning electron microscopic (SEM) images of Nano-EE at (**a**) 1000 times (**b**) 30,000 times.

After three months of storage, the particle size tended to be slightly larger [Fig. 2A (a)] and was agglomerated with a size of nearly 3000 to 6000 nm [Fig. 2A (b)]. After six months of storage, the nanoparticles were more agglomerated [Fig. 2A (c)].

#### Determination of in vitro drug release of Nano-EE by HPLC

The in vitro drug release from Nano-EE was conducted by HPLC analysis at different time intervals. Simulated gastric fluid (pH = 1.2) and simulated intestinal fluid (pH = 6.8) were selected as the medium (Fig. 2B). Simulated gastrointestinal fluids are widely used for drug delivery applications because it mimics the original fluids in the stomach and intestine. The results showed that a quick release of drugs was observed at initial hours in the presence of simulated gastric fluid (pH = 1.2), and it released about 46% of drugs after 120 min. Compared to simulated gastric fluid, the drug-releasing rate in the simulated intestinal fluid was slower. It was noted that after 240 min, about 67% of the drug was released in simulated intestinal fluid. So, it was understood that a prolonged drug release was obtained from Nano-EE, especially in the simulated gastrointestinal tract.

#### Scanning electron microscopic (SEM) analysis of Nano-EE

The morphology of Nano-EE was evaluated by using SEM at 50 µm and 1 µm scales. Figure 2C showed that the particles were scattered and slightly agglomerated. Moreover, it was understood that the particle has a spherical shape.

### Animal experiment

#### Effects of EE and Nano-EE treatment on rat body weights

**Figure 3:**
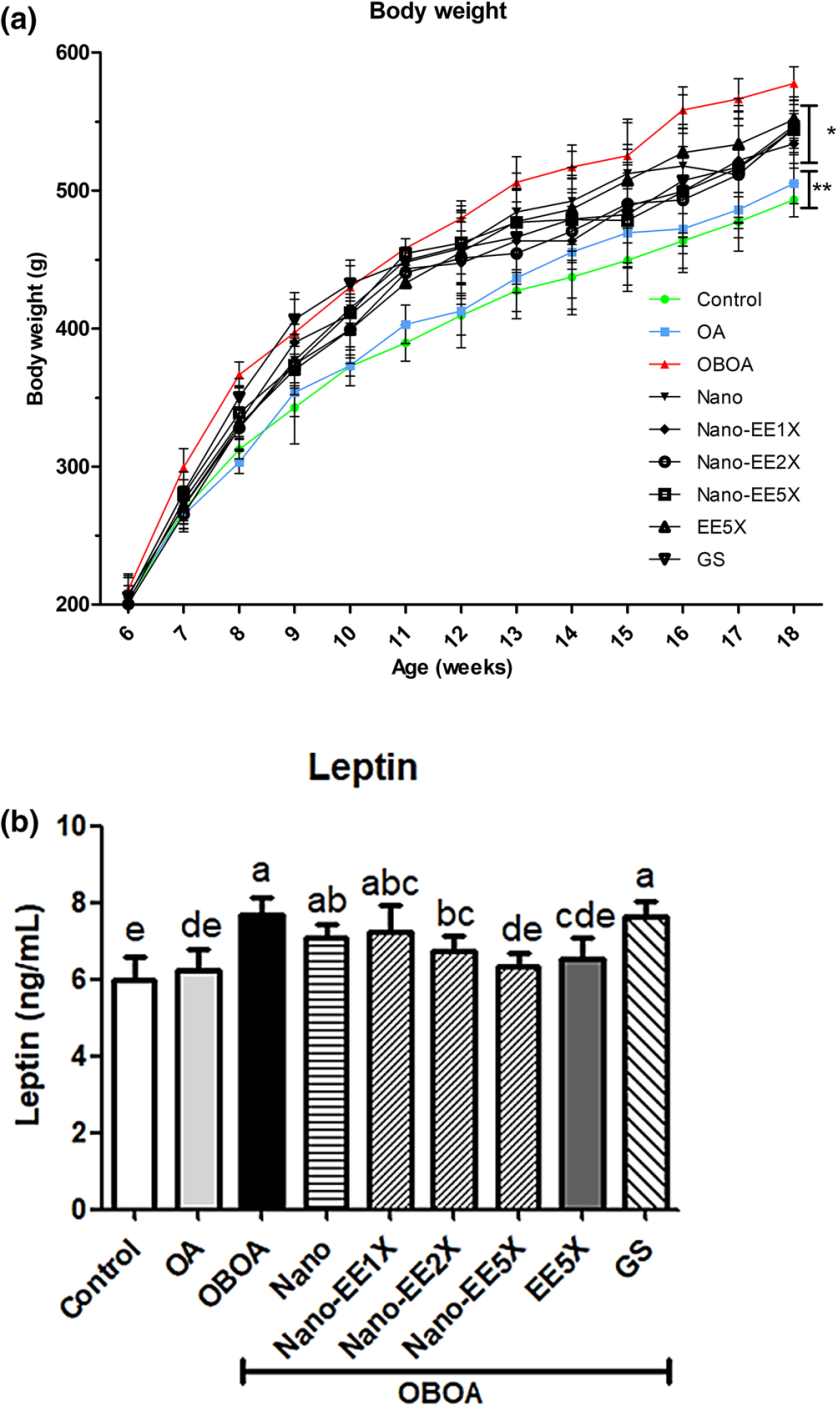
(**a**) Body weight of rats during the experiment and (**b**) Plasma leptin level in osteoarthritis rats fed different doses of Nano-EE and EE after 6 weeks. Data are shown as the mean ± SD (n = 6 rats/group). The level of leptin was analyzed by the ELISA kit. Control: sham group; OA: osteoarthritis; OBOA: obesity and osteoarthritis; Nano: obesity and osteoarthritis + 298 mg/kg per day chitosan and silica mixture; Nano-EE1X: obesity and osteoarthritis + 93 mg/kg per day Nano-EE; Nano-EE2X: obesity and osteoarthritis + 186 mg/kg per day Nano-EE; Nano-EE5X: obesity and osteoarthritis + 465 mg/kg per day Nano-EE; EE5X: obesity and osteoarthritis + 167 mg/kg per day EE; GS: obesity and osteoarthritis + 100 mg/kg per day glucosamine sulfate. P < 0.05 (*), P < 0.01 (**) vs. OBOA.

The body weight change of rats in each week was given in Fig. 3a. At the eighteenth week of age, the body weight of the OBOA group was significantly higher than that of the other groups, and the weight of the control and OA groups (fed standard chow diet) were significantly lower than the high-fat diet OA group (OBOA). There were no significant changes in the weight of the rats between Nano-EE 1X, Nano-EE2X, Nano-EE5X, EE5X, and GS groups. Compared to the above-mentioned groups, the control and OA group has lower weight. From 11 weeks onwards, the weight of the OBOA group has increased and is maintained for up to 18 weeks.

#### EE and Nano-EE treatment attenuated leptin content in osteoarthritis rats

As shown in Fig. 3B, the leptin content of the OBOA and GS groups were higher and there was no significant difference when compared to Nano and Nano-EE1X groups. Both Nano-EE2X and Nano-EE5X significantly reduced the content of leptin and have a slight difference from EE5X. There was no significant difference between Nano-EE5X and EE5X when compared to the control group.

#### Effect of EE and Nano-EE treatment on MMPs and collagen II levels in osteoarthritis rats

**Figure 4:**
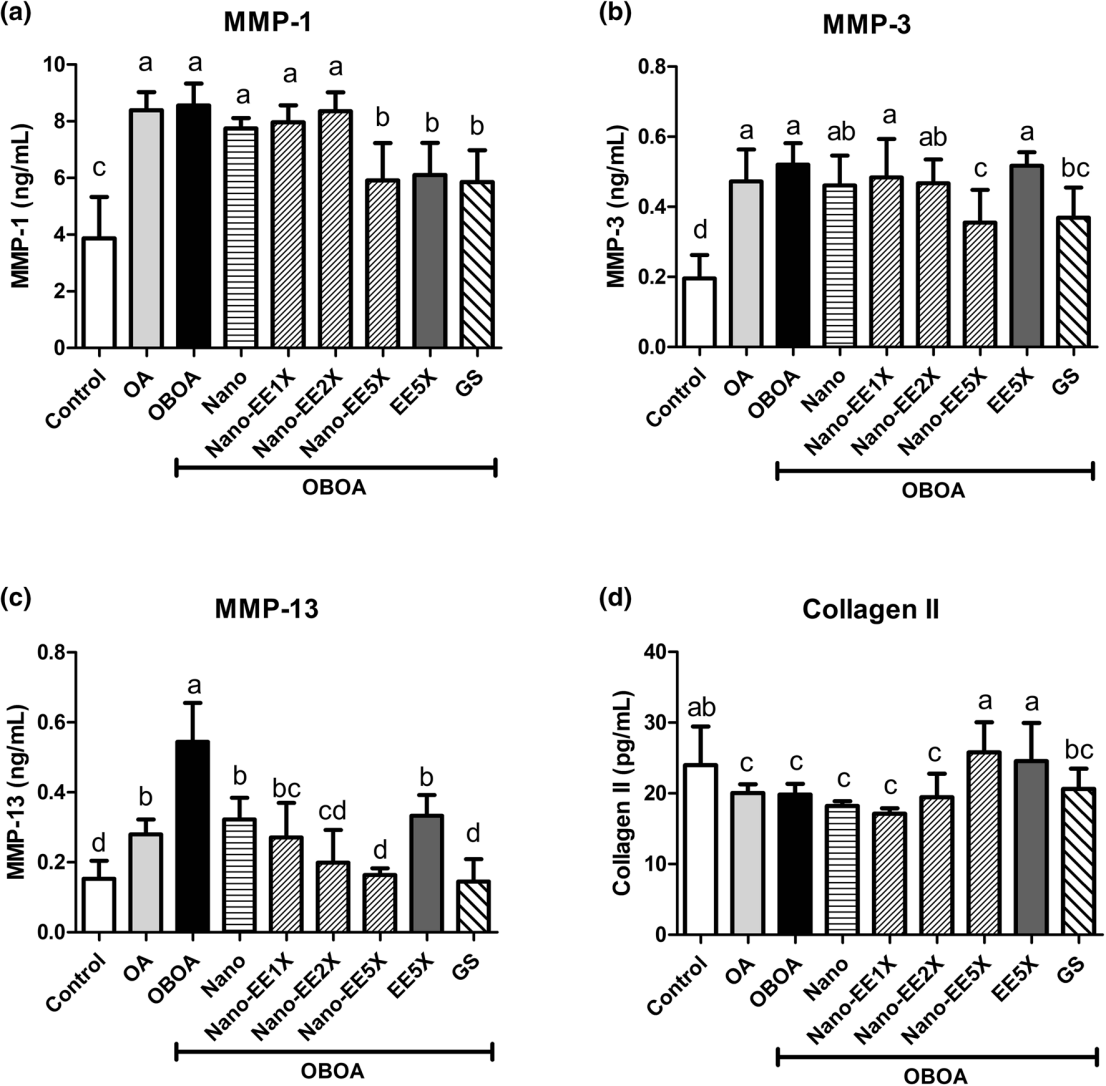
Plasma (**a**) MMP-1, (**b**) MMP-3, (**c**) MMP-13, and (**d**) type II collagen level in osteoarthritis rats fed different doses of Nano-EE and EE after 6 weeks. Data are shown as the mean ± SD (n = 6 rats/group). The levels of MMP-1, MMP-3, MMP-13, and type II collagen were analyzed by ELISA kits. The values with different letters (a–d) represent significantly different (p < 0.05) as analyzed by Duncan’s multiple range test.

The experimental results showed that the expression of MMP-1 in the OBOA group was significantly higher than in the control group. In EE5X, Nano-EE5X, and positive control groups, the expression of MMP-1 was significantly reduced when compared to other groups. (Fig. 4a). Like Fig. 4a, the level of MMP-3 was higher in the OBOA group than that of the control group. The Nano-EE5X and positive control group significantly reduced the expression of MMP-3 in the rat plasma, and the inhibitory effects of Nano-EE5X was better than the EE5X group (Fig. 4b). The MMP-13 content was also higher in the OBOA group. EE5X, Nano-EE, and the positive control groups were significantly reduced the expression of MMP-13. Besides, the MMP-13 content in Nano-EE5X was lower when compared to EE5X group (Fig. 4c). As shown in Fig. 4d, collagen II expression in rat plasma was significantly reduced in the OA-induced group. Compared with Nano-EE1X and Nano-EE2X groups, the Nano-EE5X group significantly improved Collagen II content. However, there was no significant difference between EE5X and Nano-EE5X groups. The content of collagen II in GS was lower than Nano-EE5X.

#### Effect of EE and Nano-EE treatment on inflammatory factors in osteoarthritis rats

**Figure 5:**
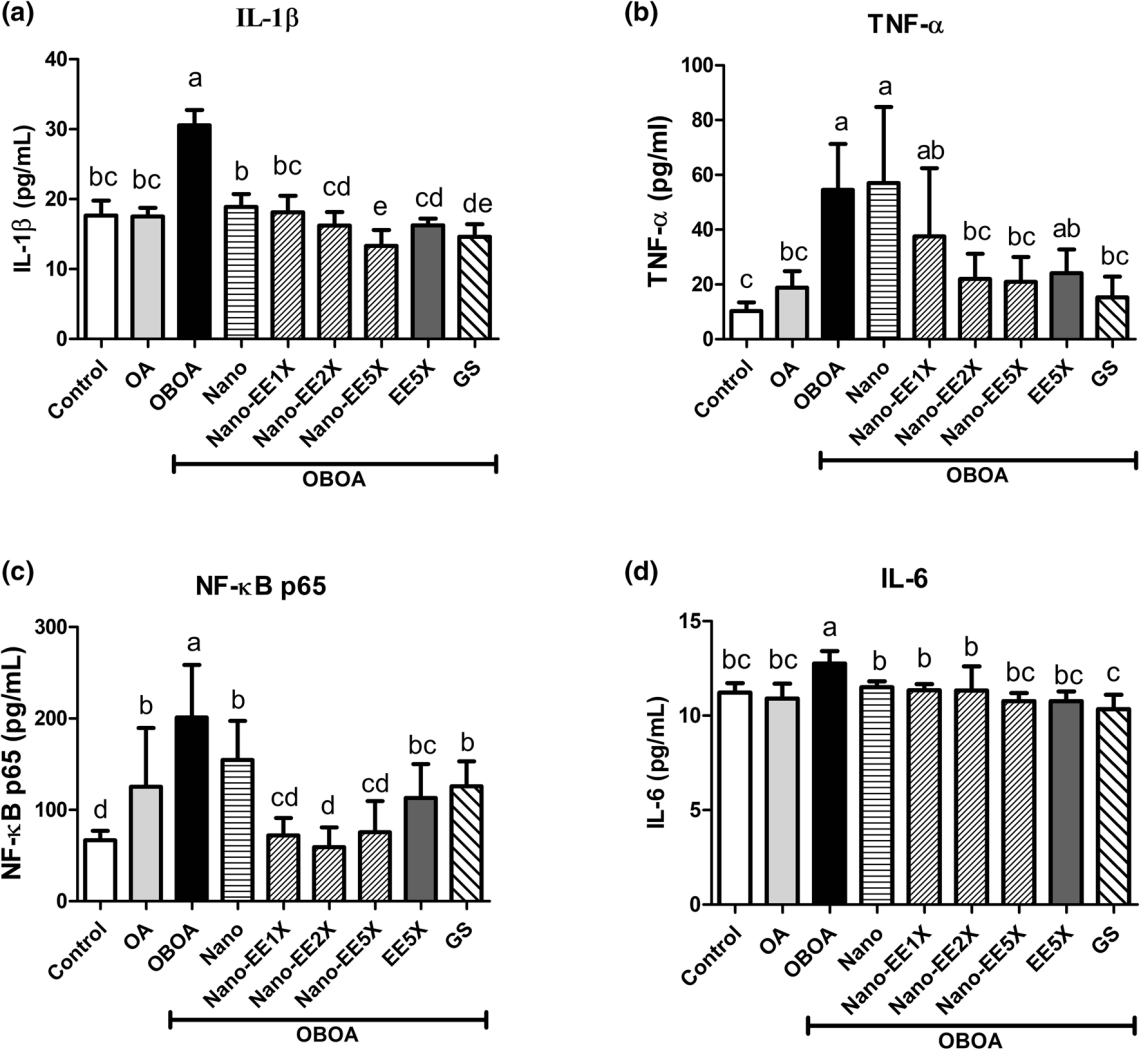
Plasma (**a**) IL-1β, (**b**) TNF-α, (**c**) NF-κB p65, and (**d**) IL-6 level in osteoarthritis rats fed different doses of Nano-EE and EE after 6 weeks. Data are shown as the mean ± SD (n = 6 rats/group). The levels of IL-1β, TNF-α, NF-κB p65, and IL-6 were analyzed by ELISA kits. The values with different letters (a–e) represent significantly different (p < 0.05) as analyzed by Duncan’s multiple range test.

The expression of IL-1β, TNF-α, NF-κB, and IL-6 in OA rats after sample treatments were given in Fig. 5. The expression of IL-1β was significantly higher in the OBOA group than other groups. A significant reduction in IL-1β level was observed after Nano-EE administration when compared to the OA and OBOA group. The highest reduction in IL-1β level was seen in the Nano-EE5X group and it was better than the EE5X group. It was also noted that the positive control group also significantly inhibited the expression of IL-1β (Fig. 5a). TNF-α expression was given in Fig. 5b. The expression of TNF-α was higher in the OBOA group along with the Nano group. So, it was understood that the Nano group did not reduce the expression level of TNF-α in rat plasma. It is also noted that the TNF-α level was increased under obesity conditions. Nano-EE2X and Nano-EE5X groups reduced the expression of TNF-α and there was no significant difference when compared with the OA group (without obesity). The TNF-α level in the GS group was lower than Nano-EE5X (Fig. 5b). The results showed that the level of NF-κB P65 in the OBOA group was significantly higher than that of the other groups. After Nano-EE administration, the expression of NF-κB P65 in the rat plasma was significantly reduced, but there was no significant difference between the Nano-EE 1X and Nano-EE5X. The positive control group also reduced the expression of NF-κB P65 but the level was higher than Nano-EE groups. NF-κB P65 level in EE5X was higher than Nano-EE5X (Fig. 5c). As shown in Fig. 5d, IL-6 level was higher in the OBOA group. After giving various doses of Nano-EE and EE5X, the IL-6 level was reduced and there was a significant difference when compared to the OBOA group. A higher level of IL-6 level was observed in obesity conditions (OBOA) and it was reduced after treatment.

#### Anti-inflammatory effect of EE and Nano-EE in osteoarthritis rats

**Figure 6:**
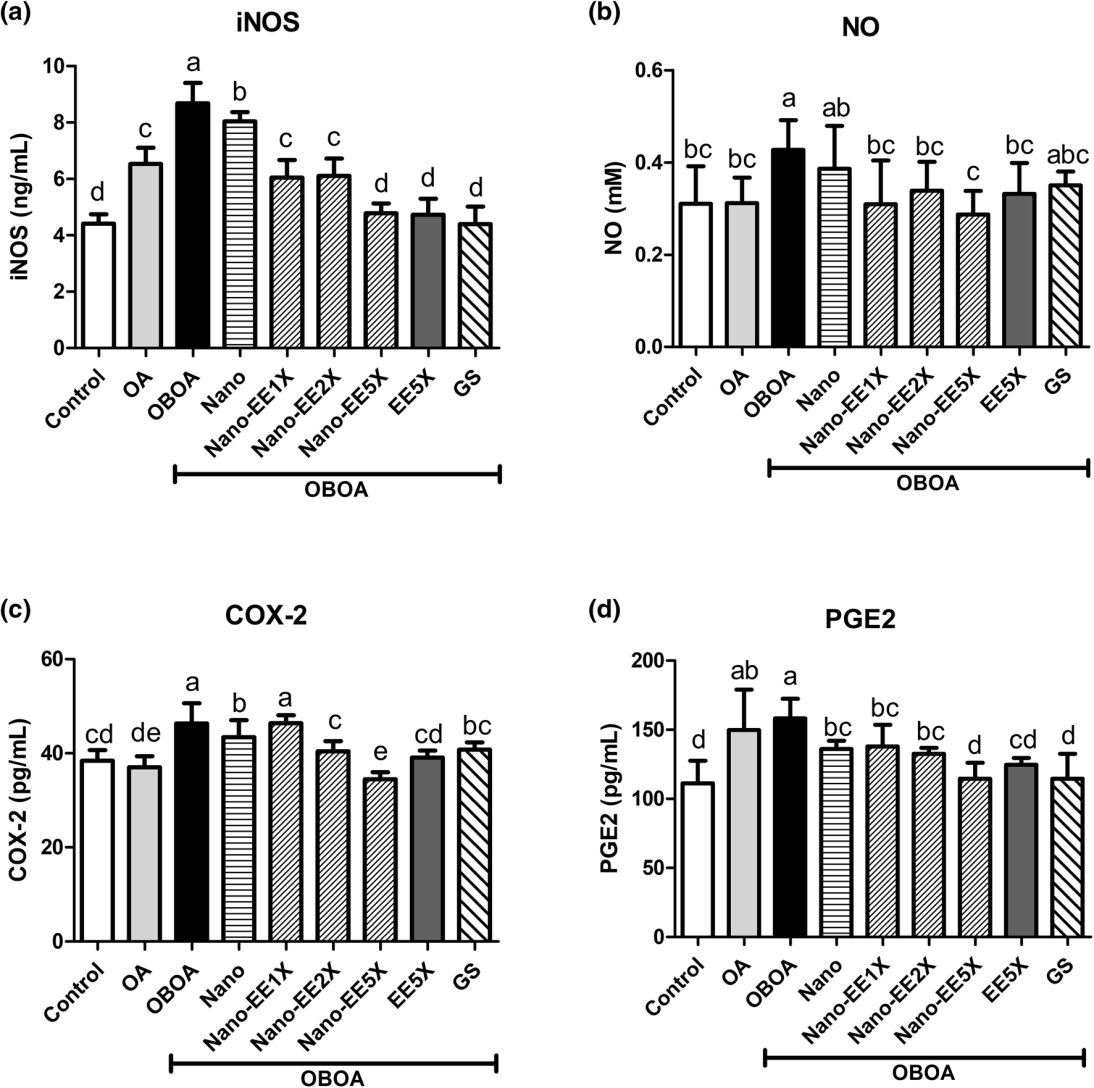
Plasma (**a**) iNOS, (**b**) NO, (**c**) COX-2, and (**d**) PGE2 level in osteoarthritis rats fed different doses of Nano-EE and EE after 6 weeks. Data are shown as the mean ± SD (n = 6 rats/group). The levels of iNOS, COX-2, and PGE2 were analyzed by ELISA kits. NO content was analyzed by the Griess reagent method. The values with different letters (a–e) represent significantly different (p < 0.05) as analyzed by Duncan’s multiple range test.

As shown in Fig. 6a, the iNOS level was higher in the OBOA group and lower in the Nano-EE groups. There were no significant differences between Nano-EE5X, EE5X, and the positive control groups. The level of iNOS in Nano-EE1X and 2X groups were higher than the above-mentioned groups (Fig. 6a). The amount of NO expressed in the OBOA group was significantly higher than that of other groups. After giving Nano-EE, it significantly inhibited the amount of NO expressed in the plasma of OA rats. NO level in EE5X was higher than Nano-EE5X (Fig. 6b). Compared to OBOA group, the Nano-EE2X group significantly reduced the level of COX-2. Nano-EE5X significantly reduced COX-2 production and it was better than EE5X groups (Fig. 6c). As shown in Fig. 6d, the PGE2 level was higher in the OBOA group, and Nano-EE groups significantly reduced the expression of PGE2. Nano-EE5X had no major difference in PGE2 level with EE5X and the positive control group.

#### Effect of EE and Nano-EE treatment on incapacitance test in osteoarthritis rats

**Figure 7:**
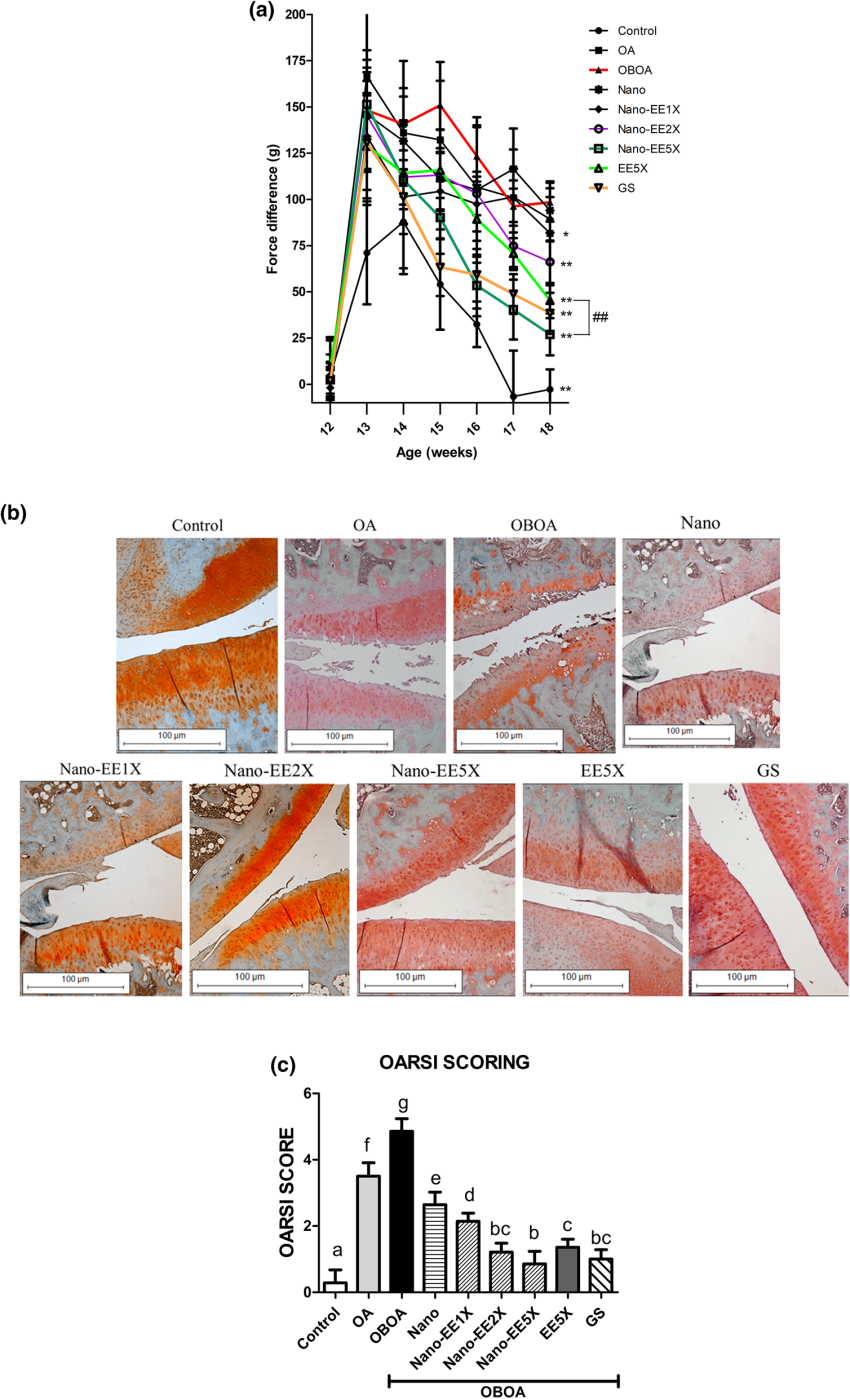
(**a**) Incapacitance test in osteoarthritis rats fed different doses of Nano-EE and EE after 6 weeks. Data are shown as the mean (n = 6 rats/group). p < 0.05 (*), p < 0.01 (**) vs. OBOA. p < 0.05 (#), p < 0.01 (##) represent significantly different between EE5X and Nano-EE5X. (**b**) Histology staining (Safranin O) of OA rats after different doses of Nano-EE and EE after 6 weeks. The scale bar length is 500 µm. The knee joints of rats with OA were stained with Fast Green/Safranin-O. (**c**) OARSI score of cartilage tissues. Grade 0: surface intact, cartilage morphology intact, grade 1: surface intact, grade 2: surface discontinuity, grade 3: vertical fissures (clefts), grade 4: erosion, grade 5: denudation, grade 6: deformation.

During the incapacitance test, before the surgery, it was found out that there was no significant difference between the groups, and the difference between the left and right incapacitance force application was very small. After a week, the surgery was conducted and the incapacitance was measured. For the control group (just cut the skin and muscle without ACLT + MMx induced OA), the values were slightly increased, but still significantly lower than the group with ACLT + MMx induced OA. In surgically induced OA group, both feet' pain measurement values were significantly higher than those in the control group, indicating that the surgically induced OA was successful. After sample gavage (six weeks later), it was found that the value of the control group had returned to a value similar to before the sham surgery period. The values of EE5X, Nano-EE5X, and the positive control group were significantly reduced, indicating that they can reduce the pain caused by OA in rats (Fig. 7a).

#### Histology staining (Safranin O) on the knees of OArats fed with different doses of Nano-EE and EE

It was understood that OA caused the loss of proteoglycan and the presence of uneven cartilage surface under the Safranin O tissue staining section. After the slice images were taken through the microscope, it was found that the control group had an obvious accumulation of proteoglycan and the cartilage surface was flat. In OA and OBOA groups, the loss of proteoglycans and the unevenness of the cartilage surface were found. In the Nano group, there were no improvements in the loss of proteoglycan, and the surface of the cartilage of Nano-EE1X and Nano-EE2X was still observed with some unevenness and proteoglycan loss. Compared with the OBOA group, the Nano-EE5X dosage had significantly improved the loss of proteoglycan and improved the unevenness of the cartilage surface. EE5X and positive control group also improved the proteoglycan loss. The results showed that Nano-EE can effectively improve the cartilage proteoglycan loss and make the cartilage surface flat (Fig. 7b). The OARSI score of cartilage tissues was given in Fig. 7c. The OARSI score was conducted according to Pritzker et al.^32^. Healthy cartilage tissues were observed in the control group followed by Nano-EEX. Healthy tissue is characterized by a smooth cartilage layer and chondrocytes were present in well-ordered zones. The tissues of Nano-EE2X, EE5X, and GS were lying between grades 1 and 2. Superficial zone intact, oedema, and cell death were observed in grade 1 tissues. The score of Nano and Nano-EE1X were observed between 2 and 3. Surface discontinuity and vertical fissures were common in grade 2 and 3 tissues. High-grade OA histopathology was observed in the OBOA group followed by the OA group. These groups were characterized by cartilage matrix loss and denudation^27,32^.

## Discussion

Obesity is a multifactorial disease known to have excess body weight and is associated with an increase in adiposity and fat. It arises from the imbalance between energy consumption and expenditure. Obesity can be affected by various risk factors including diet, age, genetics, and socioeconomic factors^33^. Knee OA is a degenerative disease related to obesity. Overweight increases the mechanical load on cartilage, cytokines production by adipose tissue, and also facilitates inflammation^34^. Literature mentioned that obesity is one of the major modifiable risk factors of OA. Mechanical and metabolic factors are responsible for the development of OA. Decreased muscle strength, increased forces about the joint, and altered biomechanics are the main cause of OA under obesity conditions^35^. 5-unit increase in BMI is related to 35% increase in knee OA^36^. High leptin content under obesity leads to cartilage degeneration^37^. Obesity is related to an increase in the levels of pro-inflammatory cytokines (such as TNF-α, IL-1β, and IL-6) and adipokines (especially leptin)^8^. In this study, the sham group (receiving CFD feeding only) was the control to observe the body weight, fat parameters, and biochemical parameters (including lipid properties and pro-inflammatory cytokines). Besides, the sham group can be defined as a control group to ensure that the operation has the same accidental effect as the real operation. Currently, there are no any hundred percentage effective remedy for treating OA and it is also noted that conventional treatments are limited to analgesics and surgical intervention^38^. Plant extracts have been used from ancient times to treat diseases. Lee et al. (2019) used *Alpinia oxyphylla* extract for the treatment of OA^39^. Besides, anti-OA effects of *Mollugo pentaphylla* extract and *Dipsacus asperoides* extract were also reported^40,41^. The protective effect of grape seed oil on OA was also reported^42^. Mani et al. (2016), used phyto-stabilized silver nanoparticles for arthritis treatment and *Piper nigrum* extract was used as a stabilizer^43^. In this study, encapsulated *Echinacea purpurea* ethanol extract was used for treating obesity-induced OA. Echinacea has been used for a long time to alleviate respiratory infections^44^. A previous study reported the reduction of NO production and inflammation in LPS-induced RAW 264.7 macrophage cells^13^.

In this study, EE was encapsulated in chitosan silica nanoparticles and the nanoparticles were characterized by dynamic light scattering, and scanning electron microscopy. DLS was used to determine the average particle size, poly dispersity index, and zeta potential of Nano-EE. The PDI value represents the degree of particle dispersion of the sample. When the PDI value was low, the particle size dispersion was more good. It means that the sample was composed of particles of similar size. When the PDI value was higher than 1, it means that the sample particle size distribution was uneven^45^. The value of the zeta potential indicates the degree of electrostatic repulsion between adjacent particles. For smaller molecules and particles, the zeta potential is higher and it indicates the stability of the particle. When the absolute value of potential is small, the attractive force may exceed the repulsive force and cause the phenomenon of particle aggregation. Therefore, colloids or particles with high dielectric potential are relatively stable, while particles with a low dielectric potential tend to aggregate or agglomerate^46^. Figure 2 showed that the particles were found to be slightly agglomerated after 3 months of storage. After six months of storage, the particles were more agglomerated. Agglomeration is due to the adhesion of particles and as a result, the surfaces become reduced and the size of the particles is increased. During agglomeration, the particles are attracted by weak forces^47^.

The in vitro drug release profile of Nano-EE by HPLC analysis at various time points in simulated gastric fluid (pH = 1.2) and simulated intestinal fluid (pH = 6.8) were also determined. It was understood that the Nano-EE has a controlled drug release under simulated intestinal fluid than simulated gastric fluid. The reason for the fastest initial release rate was due to the release of EE that adhered to the surface of the nanoparticles. Besides, the surface of the nanoparticles was composed of chitosan. Chitosan was more likely to disintegrate in an acidic solution, cause nanoshell to rupture and release the sample^48^. Furthermore, Nano-EE can delay the releasing time of EE in the gastrointestinal tract and can reduce the condition in which the active ingredients were diluted and degraded earlier in the gastrointestinal tract.

We extend the study by performing the in vivo analysis of samples. The weight of Nano-EE, EE5X, and the positive control groups were lower than the OBOA group. Higher bodyweight of the OBOA group causes more pain when walking or moving and the willingness to walk and move was lower than other groups. After the sample gavage, the right hindlimb does not cause severe pain while moving. Improvements in OA conditions resulted in a higher willingness to move and a lower body weight. Studies showed that obesity is one of the risk factors that lead to OA in the hands and wrists. Adipose tissue has a major role to promote systemic low-grade inflammation by secreting cytokines and adipokines. Besides, the level of low high-density lipoproteins (HDLs) and high level of free fatty acids (FFAs), triglycerides (TGs), and oxidized low-density lipoproteins^49^.

The performance of adipose factors in obese people was important because obesity cause a biochemical environment in which chondrocytes were not suitable for growth. For example, chondrocytes from obese OA patients had shown a different response pattern to leptin than normal or overweight patients^50^. Leptin had two possible effects on OA, one was to promote the synthesis of cartilage matrix and the performance of growth factors such as insulin-like growth factor (Insulin-like growth factor 1, IGF-1) and transforming growth factor B (Transforming growth factor β, TGF-β).Another effect was to promote the formation of bone spurs. These two effects were contradictory to the progress of OA, but increasing cartilage matrix synthesis activity observed by leptin in OA chondrocytes may occur early. Because it was characterized by chondrocyte synthesis enhanced activity^51^.

OA is characterized by excessive loss of extracellular matrix (ECM) which is composed of type II collagen and proteoglycans^7^. However, type II collagen is degraded by MMP^52^. MMP-3 and MMP-13 are the key enzymes responsible for the degradation of type II collagen^53^. MMP-3 also promotes the production of MMP-1, MMP-7, and MMP-9^54^. Therefore, measured the content of MMP to determine whether Nano-EE can inhibit the expression of MMP in rat plasma and increase the expression of type II collagen to achieve the effect of ameliorating OA. According to the results, Nano-EE can inhibit the activity of matrix metalloproteinases and increase the expression of type II collagen to improve the loss of cartilage matrix and ameliorate OA. It is noted that the level MMPs are higher in plasma and synovial fluid of patients with OA. MMP level may increase due to inflammation^55^. This result was supported by Lu et al., 2013 in which the MMP level was higher in knee OA^56^.

Several inflammatory factors such as COX-2, PGE2, and IL-6 are secreted as a result of activation of the NF-κB pathway by IL-1β and TNF-α. Literature mentioned that the cichoric acid extracted from *Echinacea purupure*a has been tested on collagen-induced arthritis (CIA) rats. The results showed that the *Echinacea purpurea* cichoric acid extract can reduce the expression of TNF-α, IL-1β, COX-2, PGE2, and NF-κB in CIA rats^57^. When the NF-κB pathway is activated, the inhibitory protein IκB-α inhibits P65 and P50 and causes their degradation in the cytoplasm. So that the phosphorylated P65 and P50 in the cytoplasm will enter the nucleus for transcription and translation to produce inflammatory reactions and inflammatory products. Based on our results, it was determined that Nano-EE inhibited the expression of IL-1β, TNF-α, NF-κB P65, and IL-6 in the NF-κB pathway.

As a result of inflammation, inducible nitric oxide synthase (iNOS) catalyzes the production of NO from L-arginine. NO stimulates the chondrocytes to produce IL-1, TNF-α, and other cytokines to cause the degradation of the cartilage^58^. Literature mentioned that COX-2 is produced by arachidonic acid and inflammatory factors (IL-1 and IL-6) as a result of an inflammatory reaction. COX-2 catalyze the formation of PGE2 from arachidonic acid and it will eventually lead to inflammatory reactions and pain^58^. Our results showed that the Nano-EE can reduce the expression of COX-2, PGE2, iNOS, and NO.

The main symptom of OA was chronic pain, but the pain was difficult to evaluate objectively because the individual's perception of pain was too subjective. This feeling will vary due to the emotional and cognitive components of pain perception. While many pain assessments in OA animal models were conducted based on behavior, the methods for assessing animal pain can be direct or indirect. Indirect measurements can include static or dynamic weight-bearing, a posture of foot placement, gait analysis when walking, and mechanical/heat/cold sensitivity. Incapacitance and knee compression tests are some of the direct methods used for analyzing the pain^59^. The results showed that EE5X, Nano-EE5X, and positive control groups had significantly lowered the incapacitance test values. The value of Nano-EE was significantly different from EE5X. Therefore, Nano-EE can more effectively reduce the pain caused by OA in rats than EE5X.

The staining of cartilage matrix (orange to red), nuclei (black), and cytoplasm (bluish or grey-green) by safranin-O staining were conducted according to another study^60^. According to this study, OARSI scoring indicated that the tissues were good in Nano-EE5X group when compared to OA and OBOA groups. The loss of proteoglycan and the degradation of cartilage might be due to the activation of the NF-κB pathway, which caused an increase in MMP expression. Besides, we observed that the surface of cartilage surface was discontinuous and the proteoglycan content was lost in the OBOA group. Nano-EE5X significantly improved the loss of proteoglycan compared with the OBOA group. The cartilage surface unevenness also significantly improved. EE5X and the positive control group also improved the condition of proteoglycan loss. The results showed that Nano-EE can effectively improve the loss of cartilage proteoglycans together with the surface.

In this study, we evaluated the protective effect of EE encapsulated EE on OA conditions. The particles were spherical and have a good drug release profile. Our in vivo results showed that Nano-EE can reduce the expression of inflammatory mediators. It reduced the production of MMPs and increased the expression of type 2 collagen in plasma. Also, Nano-EE lowered the pain and improved proteoglycan content in OBOA rats. It was confirmed that encapsulation increases the bioavailability of *Echinacea purpurea* ethanol extract in vivo. However, this study was limited to the lack of evaluation of cytokines and MMPs in the cartilage tissue and also lacks the determination cartilage score. In the future, we would like to extend the study by adding the above-said factors.

## Methods

### Preparation of *Echinacea purpurea* ethanol extract (EE)

*Echinacea purpurea* was purchased from Taiwan Direct Biotechnology Corp., Taoyuan, Taiwan, and a voucher specimen was deposited. *Echinacea purpurea* was dried and grounded. Then, 70% ethanol was added and stirred at 40 °C for 24 h to obtain the crude extract. The solution was filtered and freeze-dried (Freeze drying system FD 4.5 12XL, Kingmech Co., Ltd., Taipei, Taiwan). The samples were kept overnight at a − 80 °C refrigerator before lyophilization. The extraction yield was approximately 10%.

### Preparation of micro-nanoencapsulated *Echinacea purpurea* ethanol extract (Nano-EE)

Chitosan silica nanoparticles were prepared according to a previously reported method^25^. Chitosan solution was prepared by dissolving chitosan in 0.1 M acetic acid solution (0.7% w/w) and the silicate solution is prepared by dissolving sodium silicate in 0.05 M sodium acetate solution (0.7% w/w). The ratio of chitosan solution, sodium silicate solution, and EE solution was 10:1:1. The solution was stirred at room temperature for 30 min at a constant speed. The solution was subjected to high-speed centrifugation (6000×*g*, 30 min) (High-speed centrifuge, Hettich CR-12, Germany). Finally, the supernatant was taken for freeze-drying. The obtained powder after freeze-drying was micro-nanoencapsulated *Echinacea purpurea* ethanol extract (Nano-EE)^25,61^.

### Encapsulation efficiency (EE) and loading capacity (LC)

The encapsulation efficiency and loading capacity were determined by HPLC (JASCO, Eaton, Maryland, USA) analysis. The analysis was conducted by detecting the content of cichoric acid in Nano-EE and EE. The encapsulation efficiency and loading capacity of Nano-EE were calculated by the following formulas:

1 $${\text{Encapsulation}}\;{\text{efficiency}} = [({\text{A}} - {\text{B}})/{\text{A}} \times 100\% ]$$

A is the total amount of cichoric acid and B is free cichoric acid in the supernatant.

2 $${\text{Loading}}\;{\text{capacity}} = [({\text{A}} - {\text{B}})/{\text{C}} \times 100\% ]$$

A is the total amount of cichoric acid, B is free cichoric acid in the supernatant, and C is the weight of the nanoparticles^62^.

### Particle size, size distribution, and surface charge of Nano-EE

The Nano-EE was diluted 1000 times with deionized water and then filtered with a 0.22 µm membrane. 700 µL sample was added to the quartz tube and the average particle size and zeta potential were measured. The experiment was performed in triplicates (Malvern Instruments, Malvern, UK). The shape of the particle was determined by scanning electron microscopy (SEM). The conductive double-sided tape was attached to the surface of a test plate. Nano-EE was dissolved in 75% of alcohol. The sample was dropped on the tape and was dried in a convection oven overnight. Samples were coated with a thin layer of gold and observed under SEM (Olympus Hitachi, Tokyo, Japan)^62,63^.

### In vitro drug release

The micro-nanoencapsulated particles are dissolved in simulated gastric fluid (SGF) and simulated intestinal fluid (SIF). SGF contained 3.2 mg/mL pepsin in 0.03 M NaCl and the pH was adjusted to 1.2. SIF contained 10 mg/mL pancreatin in 0.05 M KH_2_PO_4_ and the pH was adjusted to 6.8. Nano-EE (2%) was placed in 100 mL of SGF and stirred at 37 °C. During the experiment, aliquots were taken at specific time intervals (0, 5, 15, 30, 45, 60, 90, and 120 min) and refilled with an equal volume of fresh SGF. Finally, the reaction was stopped by cooling in ice. After the SGF test, the system was centrifuged at 20,000×*g* for 30 min. The supernatant was removed and the precipitate was added to SIF at 37 °C. The aliquots were taken at specific time intervals (0, 30, 60, 90, 120, 180, and 240 min). After taking the amount of SGF, an equal volume of fresh SIF was added.

The drug release in the fluid was measured by HPLC at 330 nm. The in vitro drug release is calculated by the following equation:

3 $${\text{In}}\;{\text{vitro}}\;{\text{drug}}\;{\text{release}}\,(\% ) = {\text{AS}}/{\text{AT}} \times 100\%$$

AT is the absolute amount of EE in infinite time, AS is the amount of EE released at a specific time interval^62,63^.

### In vivo analysis

Five-week-old Sprague-Dawley (SD) male rats (Average weight, 180 g) were purchased from BioLASCO Co. Ltd., Yilan, Taiwan. The experimental rats were housed in stainless steel cages, the temperature was maintained at 23 ± 1 °C, the humidity was 40–60%, and the 12/12 light–dark cycle (07: 00–18: 59 light; 19:00–06: 59 dark) was provided. Rat fed laboratory rodent diet (Laboratory Rodent Diet 5001, PMI® LabDiet®, St. Louis, MO., USA). Food and water were provided ad libitum. The experiment was conducted after 1 week of domestication. The Institutional Animal Care and Use Committee (IACUC Approval No. 109017) of the National Taiwan Ocean University, Taiwan reviewed and approved all protocols. The experiment is approved according to the book “Guide for the care and use of laboratory animals 8th edition”, National Academies Press (2011) and AAALC (Association for the assessment and accreditation of laboratory animal care), International. After acclimatization, rats (N = 63) were randomly divided into two groups such as Sham [n = 14; feeding with chow-fed diet (CFD)] and obesity [OB; feeding with high-fat diet (HFD), n = 49] groups. The HFD is composed of ~ 20% of fat in the total diet or ~ 40% calories from fat by adding lard according to a previously reported method as shown in Table 2^64^.

**Table 2:** Chow fed diet (CFD) and high fat fed diet compositions.

Compositions (calorie, %)	Chow-fed diet^a^	High-fat diet^b^
Carbohydrate (%)	48.70 (58.00)	43.50 (40.21)
Protein (%)	23.90 (28.50)	21.53 (19.73)
Fat (ether extract, %)	5.00 (13.50	19.26 (40.05)
Fat (acid hydrolysis, %)	5.70 (0)	5.09 (0)
Fiber (%)	5.10 (0)	4.56 (0)
Mineral (%)	7.00 (0)	6.25 (0)
Calories (kcal/g)	3.36	4.33

^a^Laboratory Rodent Diet 5001.

^b^The diet formula was adapted from a previous study^64^.

The dosage of Nano-EE 1X is based on the recommended daily intake of 900 mg of Echinacea extract for adults announced by the Taiwan Ministry of Health and Welfare. Then use the human and rat dose conversion formula to get 1X dose. 93 mg/kg is set as 1X dose, 167 mg/kg is set as EE5X dose, and 298 mg/kg is set as Nano dose.

The rats were divided into nine groups. Sham group (control) and OA group were fed with only chow-fed diet. OA was induced by ACLT + MMx surgery. The obesity group (OB) fed with a high-fat diet for 6 weeks to induce obesity. Then OB group were divided into 7 groups. Each group was subjected to ACLT + MMx surgery to induce OA under obesity conditions (OBOA) (7 rats/group). OBOA group received saline (OBOA) and other six groups received different samples by oral gavage. Samples include chitosan/silica nanocarriers (Nano) (298 mg/kg),. Nano-EE, 5X EE (EE5X) (167 mg/kg), and glucosamine sulfate (GS) (100 mg/kg). Nano-EE were was administered at 1X, 2X, and 5X doses (Nano-EE1X/Nano-EE2X/Nano-EE5X) (93,186, 465 mg/kg). EE is the *Echinacea purpurea* ethanol extract (5times) and lucosamine sulfate was the positive control. After 6 weeks, CO_2_ was used to sacrifice rats. Blood and joint tissues were taken for future analysis.

### Surgically-induced OA

The surgery was performed according to a previously reported method with some modifications^65^. OA is induced by anterior cruciate ligament destruction and medial meniscus removal. First, rats were anesthetized with subcutaneous injection of Zoletil® 50 (1 g/mL) and the hair near the right knee joint was shaved. After disinfection with iodine, the skin was longitudinally cut at the knee joint to expose the ligament, and anterior cruciate ligament transection with partial medial meniscectomy surgery (ACLT + MMx) was conducted to induce OA. For the Sham group, the skin and muscles were opened but ACLT + MMx surgery was not conducted. Rinsed the wound with sterile physiological saline. The joint cavity was sutured with 4-0 Chromic catgut (Unik, Taiwan) and the skin was sutured with 3-0 nylon thread Braided silk (Unik, Taiwan). Finally, Cephalosporin antibiotic (10 mg/kg) was injected subcutaneously to prevent postoperative infection^65^.

### Blood sample collection

The blood was collected from the rat aorta using a heparinized syringe. Plasma and serum were separated from whole blood by centrifugation (3000 rpm) at 4 °C for 15 min (Kubota Centrifuge 3500; Kubota Corp., Tokyo, Japan). The supernatant was collected (plasma) and stored at − 20 °C for further analysis.

### Cytokine analysis

MMP-1, MMP-3, MMP-13, Collagen II, IL-1β, TNF-α, IL-6, and iNOS were purchased from Elabscience, Hubei, China. NF-κB p65, COX-2, and PGE2 were purchased from Taiclone (Taipei, Taiwan). All analyses were performed according to the manufacturer’s instructions. Nitric oxide (NO) production was analyzed by the Griess reagent method. 100 μL of plasma and serially diluted standard (0.1 M Nitrite) were added to the 96-well plate. Then, 50 μL of SUL solution (0.1% sulfanilamide solution in 2.5% phosphoric acid), and 50 μL of NED solution (0.1% N-(1-naphthyl) ethylenediamine dehydrate chloride in dd water) were added. After 10 min, the absorbance was measured at 540 nm. The amount of NO was calculated by plotting the standard curve^66^.

### Incapacitance test

The weight distribution of the hind limbs of the rats was measured with a incapacitance analgesia meter. The rats were trained to stand on hind legs in an acrylic box equipped with a 65° inclined plate. In the official test, the acrylic box will be installed on the biped balance tester to make the rat stand on the hind limbs. The biped balance tester will measure the pressure of the two hind limbs, and the average value will be taken after five times repeat^67^.

### Histopathology analysis and OARSI scoring

After 48 h of fixation with 4% paraformaldehyde, the knee joint was immersed in EDTA (20% EDTA, pH = 7.4) for several days for decalcification. After the decalcification is completed, the tissue is embedded with paraffin and cut into 5 μm thick slices. Subsequently, randomly selected slices were stained with Safranin-O to observe the loss of proteoglycans in cartilage^68^. The OARSI scoring was conducted according to Pritzker et al.^32^. Grade 0: surface intact, cartilage morphology intact, grade 1: surface intact, grade 2: surface discontinuity, grade 3: vertical fissures (clefts), grade 4: erosion, grade 5: denudation, grade 6: deformation.

### Ethics approval and informed consent

All methods were carried out in accordance with relevant guidelines and regulations. The Institutional Animal Care and Use Committee (IACUC Approval No. 109017) of the National Taiwan Ocean University reviewed and approved all protocols. The experiment is approved according to the book “Guide for the care and use of laboratory animals 8th edition”, National Academies Press (2011) and AAALC (Association for the assessment and accreditation of laboratory animal care), International. The study was carried out in compliance with the ARRIVE guidelines.

### Statistical analysis

The statistical analysis of the data is performed by Graph Pad Prism 5 (URL: https://www.graphpad.com/) and Statistical Product & Service Solutions (SPSS) 22.0 software (URL: https://www.ibm.com/in-en/analytics/spss-statistics-software). Statistical differences were analyzed by one-way analysis of variance (One-way ANOVA), and differences between groups were compared by Dunnett's Test for post-hoc comparison. When p < 0.05 means a statistically significant difference.

## Data Availability

The data presented in this study are available on request from the corresponding author.
